# Mimosoid legume plastome evolution: IR expansion, tandem repeat expansions, and accelerated rate of evolution in *clpP*

**DOI:** 10.1038/srep16958

**Published:** 2015-11-23

**Authors:** Diana V. Dugas, David Hernandez, Erik J.M. Koenen, Erika Schwarz, Shannon Straub, Colin E. Hughes, Robert K. Jansen, Madhugiri Nageswara-Rao, Martijn Staats, Joshua T. Trujillo, Nahid H. Hajrah, Njud S. Alharbi, Abdulrahman L. Al-Malki, Jamal S. M. Sabir, C. Donovan Bailey

**Affiliations:** 1 Department of Biology, New Mexico State University, P.O. Box 30001, MSC 3AF, Las Cruces, NM, 88003, USA; 2 Institute of Systematic Botany, University of Zurich, Zollikerstrasse 107, 8008 Zurich, Switzerland; 3 Department of Integrative Biology, The University of Texas at Austin, 205 W. 24th St. Stop C0930, Austin, TX 78712, USA; 4 Department of Biology, Hobart and William Smith Colleges, 300 Pulteney Street, Geneva, NY 14456, USA; 5 Oregon State University, Department Of Plant Biology, 2082 Cordley Hall, Corvallis, OR, 97331, USA; 6 Biosystematics Group, Wageningen University, Droevendaalsesteeg 1, 6708 PB, Wageningen, The Netherlands; 7 Biotechnology Research Group, Department of Biological Sciences, Faculty of Science, King Abdulaziz University, Jeddah 21589, Saudi Arabia; 8 Department of Biochemistry, Faculty of Science, King Abdulaziz University, Jeddah 21589, Saudi Arabia

## Abstract

The Leguminosae has emerged as a model for studying angiosperm plastome evolution because of its striking diversity of structural rearrangements and sequence variation. However, most of what is known about legume plastomes comes from few genera representing a subset of lineages in subfamily Papilionoideae. We investigate plastome evolution in subfamily Mimosoideae based on two newly sequenced plastomes (*Inga* and *Leucaena*) and two recently published plastomes (*Acacia* and *Prosopis*), and discuss the results in the context of other legume and rosid plastid genomes. Mimosoid plastomes have a typical angiosperm gene content and general organization as well as a generally slow rate of protein coding gene evolution, but they are the largest known among legumes. The increased length results from tandem repeat expansions and an unusual 13 kb IR-SSC boundary shift in *Acacia* and *Inga*. Mimosoid plastomes harbor additional interesting features, including loss of *clpP* intron1 in *Inga*, accelerated rates of evolution in *clpP* for *Acacia* and *Inga*, and *dN/dS* ratios consistent with neutral and positive selection for several genes. These new plastomes and results provide important resources for legume comparative genomics, plant breeding, and plastid genetic engineering, while shedding further light on the complexity of plastome evolution in legumes and angiosperms.

Legumes (Leguminosae) represent one of the most ecologically diverse and economically important plant families, with many of them producing protein-rich plant products (seeds, leaves, roots, etc.) via symbioses with nitrogen-fixing bacteria^1^,^2^,^3^. As a result of these qualities, considerable research has been conducted on legume biology over many decades^4^. Recent advances in next generation sequencing (NGS) have massively advanced research on legume comparative genomics^5^, providing a growing understanding of the basic biology of legumes and new tools for genome-enabled cultivar improvement (Legume Information System, LIS, http://legumeinfo.org/)^6^.

Of great interest here is characterization of legume plastid genomes (plastomes) in terms of the conserved and unique elements of gene content, overall structure, and the complex functional interactions with thousands of nuclear-encoded genes that once resided in the ancestral plastid genome^7^,^8^. Angiosperm plastomes most often retain an ancestral complement of genes and an organization that includes the “large single copy” (LSC), “small single copy” (SSC), and “inverted repeat” (IR) regions. Typical plastome sizes range from 120–160 kb^8^,^9^, but several recently described plastomes fall well outside these norms and/or show considerable structural rearrangement^8^. Thus, despite the commonly held view of plastomes maintaining conserved structure and sequence, recent and historical studies remind us that some lineages harbor considerably more variation^8^,^10^,^11^.

Members of several families, including Campanulaceae, Caryophyllaceae, Ericaceae, Geraniaceae, Leguminosae, and Lobeliaceae, provide examples of groups known to harbor considerable atypical plastome variation^10^,^12^,^13^,^14^,^15^,^16^,^17^,^18^,^19^. This variation likely derives from several common mechanisms^10^, prompting research on the plastomes of these lineages to understand both the shared and unique mechanisms responsible for novel features. The Leguminosae represent one such lineage that is emerging as a model system to investigate aspects of plastome evolution.

Structural variation among legume plastomes was originally detected using restriction site and gene mapping studies and has continued through the sequencing of complete plastomes (e.g., Table 1). Examples of variation within the family include several large inversions^20^,^21^,^22^, the remarkable loss of the IR in the “inverted repeat lacking clade” (IRLC) of papilionoid legumes^23^, shifts in the rate of mutation^10^,^24^,^25^, losses of *accD*, *infA*, *rps16*, and *rpl22* genes^6^,^26^,^27^,^28^, and at least two parallel losses of *clpP* intron 1^29^.

Given this growing abundance of data on variation among legume plastomes, one might expect that there is little to be gained from sequencing additional legume plastomes. However, the available legume plastid genome data are almost exclusively from closely related members of the subfamily Papilionoideae, which are among the most important as human food, livestock feed, and nitrogen fixation (e.g., *Glycine, Lathyrus, Lupinus, Medicago, Phaseolus, Pisum, Trifolium* and *Vigna*). Little comparative analysis of mimosoid or caesalpinoid plastomes is available^28^, meaning that investigation of legume plastomes has essentially focused just on the ‘tip of the iceberg’^1^ in terms of the family as a whole.

It is notable that the available legume plastomes show no significant expansions of IR regions or major variation in tandem repeat content. IR expansion is the major contributor to the largest known plastome, *Pelargonium* x *hortorum*^30^ and is well known in numerous other angiosperm families^31^. The importance of tandem repeats to plastome size variation and structure is also poorly understood. Solanaceae represents one of the few examples where such elements are an important contributor to plastid genome size variation^32^.

Subfamily Mimosoideae comprises around 83 genera and ca. 3,300 species, distributed pantropically, spanning all the major lowland tropical biomes - tropical rain forests, seasonally dry forests, deserts and savannas, and comprises a wide range of mainly woody perennial growth forms including giant trees, small trees, lianas, woody shrubs, functionally herbaceous geoxylic subshrubs, but also a handful of species which form herbaceous perennials and aquatic herbs^2^,^3^,^33^. Although subfamily Caesalpinioideae has more genera, mimosoids are more species-rich reflecting the occurrence of several large genera and notably *Acacia* s.str. (1000+ species)^34^, *Mimosa* (ca. 540 spp.)^35^,^36^ and *Inga* (ca. 300 spp)^37^. While few mimosoids are major human food crops, the subfamily includes economically important tropical timber trees and many nitrogen-fixing trees widely used for forage, green manure, poles, firewood and other products in diverse tropical agricultural and especially agroforestry systems^38^. This importance is exemplified by the genera *Leucaena*, *Inga*, *Acacia* and *Prosopis* which are the focus of this study - all of these are prominent in tropical agroforestry^37^,^39^, and some (e.g., *Leucaena leucocephala*) have escaped to become important invasive species^40^.

In association with ongoing investigations on the evolutionary history of mimosoid legumes^3^ and other comparative genomic work, we have sequenced and assembled the plastid genomes of *Inga leiocalycina* and *Leucaena trichandra.* Using these new plastome sequences along with the recently published *Prosopis glandulosa*^28^ and *Acacia ligulata*^27^ plastomes, we characterize mimosoid-specific variation, report the characteristics of each plastome and discuss the results of comparative analyses focused on genome structure, size, and repeat contents, as well as patterns of mutation in protein coding genes.

## Results and Discussion

### Plastome Assemblies and Gene Content

**Figure 1:**
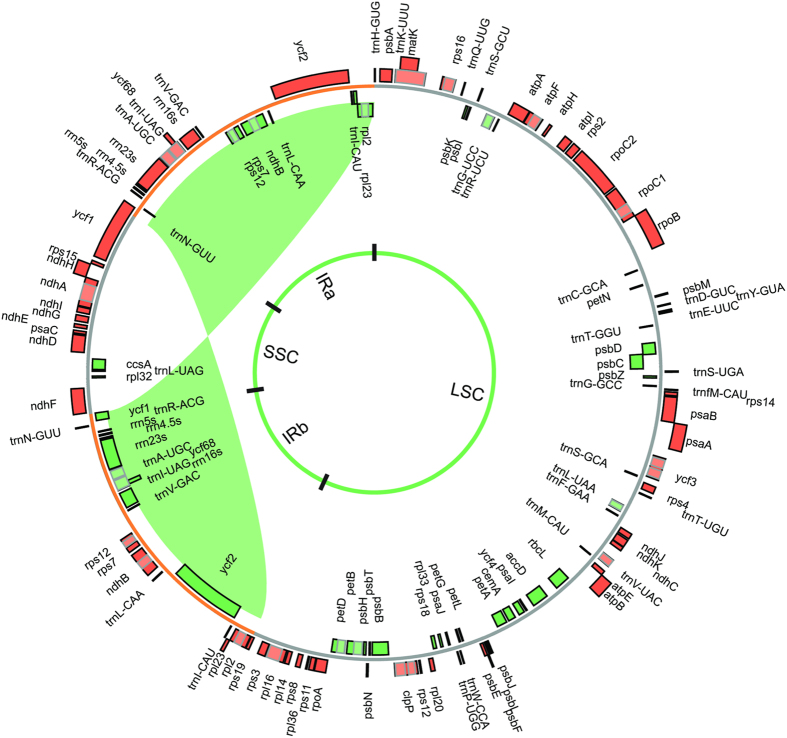
Plastid genome of *Leucaena*. Genes are indicated by boxes on the inside (green, clockwise transcription) and outside (orange, counterclockwise transcription) of the outermost circle. The inner circle identifies the major structural components of the plastome (LSC, IRs, and SSC) and the IR region is indicated by the inverted green ribbon.

Knowing that legume plastomes can harbor both large- and small-scale structural rearrangements relative to typical angiosperms, we employed a combination of reference guided and *de novo* assembly strategies (see “Materials and Methods”) for Illumina PE library-based assemblies. The reference guided assemblies for *Inga* and *Leucaena* were complicated by problems around the IR boundaries and lower coverage across some extensive repeat regions, leading us to focus on *de novo* assembly strategies (see “Materials and Methods”) to avoid possible bias imposed by the constraint of a reference. The *de novo* assemblies for *Inga, Leucaena*, and *Prosopis* were developed independently in three different laboratories prior to the development of this collaboration. The *Leucaena* plastome exemplifies most of the features found in common in the three newly sequenced genomes (Fig. 1). In each, a conserved gene order with the ancestral angiosperm^8^ and the recently published *Acacia* plastome^27^, was recovered. Furthermore, these plastomes retain the ancestral organization of angiosperms, with the typical LSC, IR, and SSC organization (but see “*Inga* IR Expansion” below).

Gene content across the mimosoids was largely conserved with the majority of other angiosperms. These plastomes each have 112 unique genes, including four ribosomal, 30 tRNA, and 78 unique protein coding genes. Each lacks the *rpl22* and *infA* genes, known to have undergone a transfer to the nucleus in other legumes^41^,^42^. The *Inga* plastome lacks the *clpP* intron 1, a finding consistent with Jansen *et al.*^29^, confirming parallel loss of this *clpP* intron with members of the papilionoid IRLC. Recently, Williams et al. (2015) demonstrated that the *Acacia clpP* sequence has an accelerated rate of synonymous and non-synonymous mutations, leading to the suggestion that at least some mimosoid taxa may have a functional nuclear-encoded copy of this gene.

### Plastome Size Variation and Repeat Content

Table 1 shows the sizes of the LSC, IRs, SSC, and full plastome for representative non-legume rosids, papilionoids, and the newly sequenced mimosoid plastomes. After the exceptionally large and rearranged *Pelargonium* plastome (218 kb)^30^, the *Inga* (175 kb), *Acacia* (174 kb), *Leucaena* (165 kb), and *Prosopis* (163 kb) plastomes are sequentially the next largest among these rosids.

**Figure 2:**
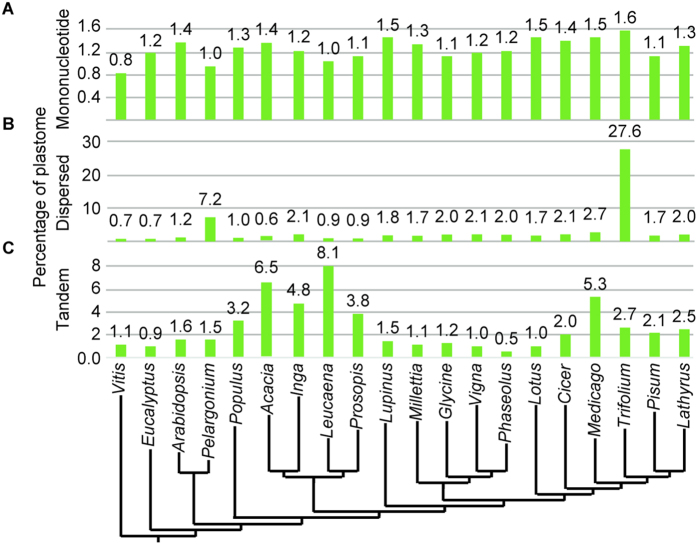
Plastome repeat content. The percentage of each plastome occupied by nucleotides recovered from: (**A**) mononucleotide, (**B**) dispersed (>16 bp), and (**c**) tandem repeats. The arrangement of plastomes is based on the currently understanding of phylogenetic relationships among these taxa.

The mimosoid LSC regions are 5–13 kb larger than other IR-containing legumes and 1.5–32 kb larger than the non-legume rosids, suggesting that much of the plastome size increase involves the LSC region. Given that changes in gene content (see above) do not account for the length increase, we investigated other likely sources. Through the plastome assembly process it became clear that AT-rich repeats were prevalent in the three new mimosoid plastomes, prompting more detailed investigation of the total number and percentage of each plastome occupied by mononucleotide, dispersed (>16 bp), and tandem repeats (Fig. 2).

These analyses recovered just 2-fold variation in the percentage of mononucleotide repeats (0.8% in *Vitis* to 1.6% in *Trifolium*) (Fig. 2A). Overall, there is little size variation attributable to mononucleotide repeats across these rosids and just 0.5% variation among the other legume samples (1.0–1.5%; Fig. 2A).

In contrast, dispersed repeats, previously discussed as important contributors to plastome size and structural evolution in *Trifolium*^10^ and Geraniaceae^30^,^43^, revealed 39-fold variation (0.7% in *Eucalyptus* to 27.6% in *Trifolium*) in percentage of plastome occupancy (Fig. 2B). However, with the exception of the extreme prevalence of dispersed repeats in *Trifolium*, legume plastomes harbored just 4.5-fold variation (0.6–2.7% in *Acacia* and *Medicago*, respectively) and only 0.6–2.1% among mimosoids. Thus while dispersed repeats are important in the evolutionarily derived *Trifolium* plastome, they apparently play only a minor role in plastome size variation across other legumes.

**Figure 3:**
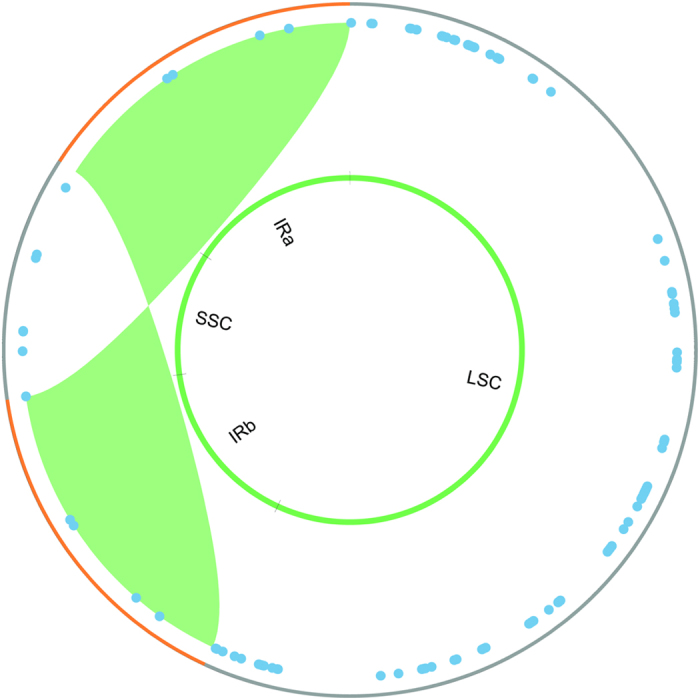
Tandem repeat distribution in the *Leucaena* plastome. Each tandem repeat is plotted as a blue dot by starting position in the plastome; repeats are not scaled by size. The inner circle identifies the major structural components of the plastome.

Among all rosids sampled, the proportion of plastomes occupied by tandem repeats is greatest within the legumes (0.5–8.1% in *Phaseolus* and *Leucaena,* respectively) (Fig. 2C), with two divergent legume lineages showing increases in tandem repeat content relative to the outgroups or other legumes. These include the mimosoid legumes (3.8–8.1%) and the IRLC papilionoids (2.0–5.3%) (Fig. 2C). By mapping the distribution of tandem repeats across the *Leucaena* plastid genome, as an example of their distribution in mimosoid plastomes (Fig. 3), we found that they are concentrated in the LSC region in *Acacia*, *Inga*, *Leucaena*, and *Prosopis*. Mimosoids had mean of 118 (±47) LSC associated tandem repeats with a mean content of 7,413 (±3,264) bp. *Leucaena* presents the most extreme example, with 91% of 13.2 kb in tandem repeats in the LSC region. Furthermore, *Leucaena* has 26 different tandem repeat sections ranging from 100–306 bp in length, explaining the difficulties encountered during initial plastome assembly. The slightly smaller *Prosopis* and *Inga* LSC regions still had at least 80% of the total tandem repeat length localized to the LSC while *Acacia* had 69%. In contrast, a sample of 10 rosid relatives had a mean of just 44 (±21) LSC-associated tandem repeats whose mean content was just 1,700 (±1,237) bp. Papilionoids retaining the IR were even more limited in LSC-associated tandem repeat content (mean of 37 [±11] repeats with 1,208 [±495] LSC-associated bp).

Furthermore, strict characterization of these extensive mimosoid tandem repeats underestimates the size of the associated low complexity regions surrounding them. For example, in *Leucaena* the largest tandem repeat (308 bp consisting of 22 copies of a 14-bp AT-based repeat) occurs within an 86% AT-rich 1.6 kb spacer between the *trnT*-UGA and *trnL*-UAA genes (with a similar ca. 1.25+ kb region in *Acacia*, *Inga* and *Prosopis*). These contrast with the same region in the related *Populus* and *Lupinus* plastomes, both of which have a short tandem repeat in the region, but an intergenic spacer of less than 500 bp. Thus these mimosoid plastomes include clearly identifiable tandem repeat expansions (e.g., 308 bp) as well as more nebulous low complexity regions (e.g., 1.3 kb) that may derive from degrading tandem repeats or other AT-rich features.

Previous reports suggest that tandem repeats play an important role in plastome size evolution in other angiosperm lineages, including *Capsicum*^32^ and *Silene*^44^. A full understanding of expansions and contractions of tandem repeat content among legumes awaits sampling of additional lineages. However, given that the mimosoid plastomes clearly contain greater tandem repeat content than other rosid plastomes or IR-containing legume plastomes, the current pattern is best interpreted as an expansion of tandem repeats within the mimosoids (and perhaps in the IRCL papiloinoids). Schwarz *et al.*^28^ have recently investigated LSC contraction in the papilionoid legumes, finding that the LSC has reduced intergenic spacer content. Those findings along with our interpretation of expansion of tandem repeats in mimosoids and the IRLC suggest that gains as well as losses of tandem repeats are playing an important role in legume plastome size evolution.

### IR boundary shifts and expansion in *Acacia* and *Inga*

**Figure 4:**
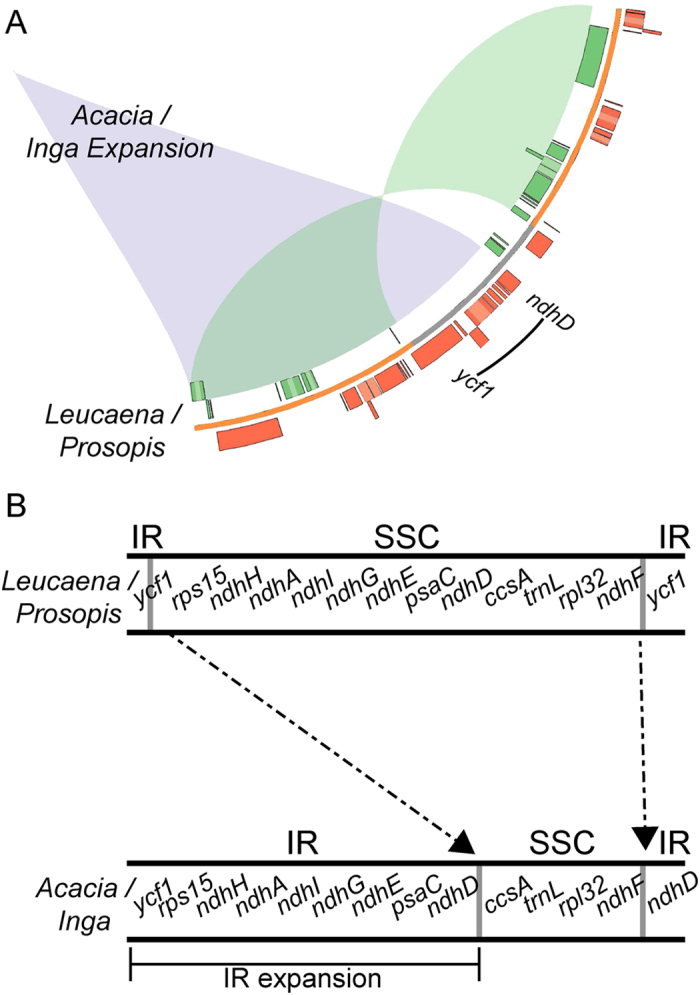
The inverted repeat expansion in *Inga* and *Acacia*. (**A**) The plastomes of *Leucaena* and *Prosopis* share similar IR boundaries, while the *Inga* and *Acacia* IRs are ca. 13 kb larger. The genic region depicts the *Leucaena*/*Prosopis* arrangement. The grey and orange lines represent the IR and SSC regions, respectively. The purple ribbon indicates the IR expansion into the SSC found in the *Acacia* and *Inga* plastomes; the green ribbon indicates the IR of the *Leucaena* and *Prosopis* plastomes. (**B**) An enlarged view of the expansion, comparing IR boundaries.

While tandem repeat expansions in the LSC have contributed to the large size of the *Inga* and *Acacia* plastomes, these genomes (the largest legume plastomes documented to date) also have IRs ca. 13 kb larger, and an SSC correspondingly smaller, than other legumes (Table 1). The large *Inga* and *Acacia* IRs are primarily the result of an extension of the IRs to include much of the SSC region (Table 1 and Fig. 4). Characterization of these boundaries revealed that the *Inga* and *Acacia* IRs are 39.8 kb and 38.2 kb, respectively, and include nine genes normally residing in the SSC (*ndhD, psaC, ndhE, ndhG, ndhI, ndhA, ndhH, rps15,* and *ycf1*) (Fig. 4). This is well outside the normal size for angiosperms, where IRs range from 20–27 kb, and the other legume plastomes that contain the IR have quite a narrow IR size range (25,156–26,481 bp, Table 1). The *Acacia* plastome also shows a further rearrangement of the IR. Not only does it include the large IR/SSC shift, it also possesses a smaller LSC/IR shift, with. 2.5 kb IR sequence in the other mimosoids being found in the LSC of *Acacia*. As a result the LSC/IR boundary in *Acacia* occurs between *rpl23* and *trnL* rather than within *rps19* where it is located in the other three mimosoids.

The detailed evolutionary history of these IR shifts remains unknown. However, given that the large IR/SSC shift is present in both the *Acacia* and *Inga* plastomes, and that *Acacia s.s*. is nested within the large tribe Ingeae (LPWG, 2013), it seems possible that the IR expansion is potentially shared across the whole Ingeae + *Acacia* clade, which comprises ca. 33 genera and ca. 2,000 of the 3,200 species of mimosoids (LPWG, 2013). Further work will be required to ascertain the extent of occurrence of these IR shifts within this large clade and whether any mimosoids outside this clade have expanded IRs.

IR expansions are well known in *Pelargonium* (75 kb)^30^, *Nicotiana acuminata* (12 kb)^45^, in the lineage that includes Campanulaceae, Lobeliaceae, and Cyphiaceae^12^, and have been recently reported in *Mahonia bealei* (12 kb)^46^ and the Trochodendraceae (ca. 4 kb)^47^. However, they are more commonly associated with extensions into the LSC rather than the SSC. The association of IR expansions with extensive poly A tracts has led to previous suggestions that poly A regions may play an important role in IR expansion^45^. The *Inga* IR extension ends in a region between *ndhD* and the SSC, which is over 78% AT-rich with numerous possible poly A tracts that may have played a role in the expansion.

### Protein coding gene rate variation

#### Rate variation in clpP and other genes

The *clpP* gene codes for a caseinolytic peptidase involved in plastid protein metabolism. Current evidence suggests that it may be essential for photosynthetic function, but not for basic cell viability in some lineages^48^. Jansen *et al.*^29^ previously demonstrated that *clpP* intron 1 is missing from 91 sampled members of the species-rich IRLC along with one (*Inga punctata*) of 18 mimosoids, identifying a pattern of parallel *clpP* intron 1 loss within the legumes. Recently, Williams *et al.*^27^ found that while retaining the intron, the *Acacia clpP* CDS has undergone a high rate of mutation. While the coding region retains the open reading frame, a variety of factors suggested that *clpP* in *Acacia ligulata* is a possible pseudogene^27^.

**Figure 5:**
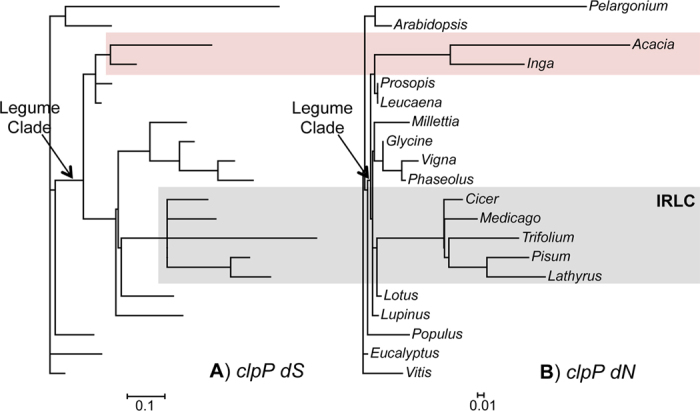
Branch lengths for *clpP*. Branch lengths were calculated on the tree representative of the current understanding of relationships for these taxa using PAML v4.7^49^. (**A**) *dS* for *clpP* and (**B**) *dN* for *clpP*. Scales are substitutions per site. Colored boxes indicate the mimosoid (red) and IRLC (grey) lineages that harbor high dN/dS values.

Intron presence/absence, branch lengths, and *dN/dS* ratios among the plastomes analyzed here shed new light on the evolution of *clpP* in legumes. The *Inga*, *Leucaena,* and *Prosopis* plastomes also retain the *clpP* open reading frame, but *Inga* has lost the intron. Furthermore, the *dN* branch lengths subtending and within the *Acacia* + *Inga* clade (Fig. 5) are long compared to all the other legumes except for those associated with the IRLC (Fig. 5), which is a group known to lack the *clpP* intron and for its rapid *clpP* divergence^29^. Previously, Williams *et al.*^27^ found little signal of selection along an *Acacia* only terminal branch (*dN/dS* = 1.07), supporting the idea that the plastome-encoded *clpP* in *Acacia* may be a pseudogene. Our *dS* (Fig. 5A) and *dN* (Fig. 5B) values derived from PAML^49^, using the aligned *clpP* data and a prior established legume phylogeny^3^, are in line with the findings for *Acacia* (*dN/dS* = 1.05), but the ratio was skewed toward more rapid change on the non-synonymous side in its sister lineage *Inga* (*dN/dS* = 1.69). Perhaps more importantly, the branch subtending the *Acacia* plus *Inga* clade recovered a *dN/dS* of 3.03, suggesting that *clpP* may have experienced positive selection. Thus, the combination of *dN/dS* ratios and the retention of a 600+ bp stop codon-free CDS in a lineage spanning millions of years of evolutionary time suggest that this gene has undergone rapid change while likely remaining under functional constraint in these lineages.

With the exception of the IRLC *clpP* lineage, which has some high *dN/dS* values and a parallel pattern of rapid mutation (Fig. 5A,B), the remainder of the rosid tree showed *clpP dN/dS* ratios of less than 0.40 (Suppl. Fig. 1C) and shorter branch lengths. These legume-wide findings mirror patterns seen in the caryophyllid angiosperms^44^, in which parallel events of accelerated mutation and relaxed and/or positive selection on *clpP* in derived lineages are clearly evident.

Similar instances of rate increases and/or *dN/dS* ratios >1 were detected for several other mimosoid genes. These include the *Leucaena* and *Propospis* clade in *atpF* (Suppl. Fig. 1A), the *Leucaena* plus *Prosopis* clade and *Inga* for *cemA* (Suppl. Fig. 1B), the *Leucaena* terminal in *psbH* (Suppl. Fig. 1C) and p*sbT* (Suppl. Fig. 1D), as well as *rps2* (Suppl. Fig. 1E), *rps3* (Suppl. Fig. 1F), and *rps4* (Suppl. Fig. 1G) for various mimosoid branches. Similar findings were first uncovered in *Pelargonium*, where rate shifts led Guisinger *et al.*^50^ to conclude that a combination of DNA repair and gene expression differences might drive such high rates of nucleotide substitution. Accelerated rate variation has since been observed for genes from related functional groups in other angiosperm lineages, including Apocynaceae (*clpP*)^51^, Caryophyllales (*rps* and *clpP* genes)^16^,^44^, Poaceae (*psb* genes)^52^, and Saxifragales (*rps* genes)^53^. As discussed by Guisinger *et al.*^50^ and Sloane *et al.*^44^, these recurrent patterns of unusual rate variation in genes from similar functional groups are intriguing and likely result from interrelated convergent factors. This idea is further supported by correlated elements of divergence in the plastid and mitochondrial genes from some of these same lineages, suggesting that parallel changes in DNA replication, repair, recombination, and/or levels of expression are playing important roles in plant organellar evolution^16^,^50^,^54^. Some of these mechanisms may also be important in the expansion of mimosoid tandem repeats (discussed above). Given that the legumes are one of the families with the greatest diversity of parallel acceleration of mutation in gene sets, and that they harbor a wide range of structural rearrangements and a wide range of repeat expansions and contractions, the family does indeed deserve attention as a model system for understanding the underlying mechanisms of plastome evolution.

#### Substitutions vs. indels

**Figure 6:**
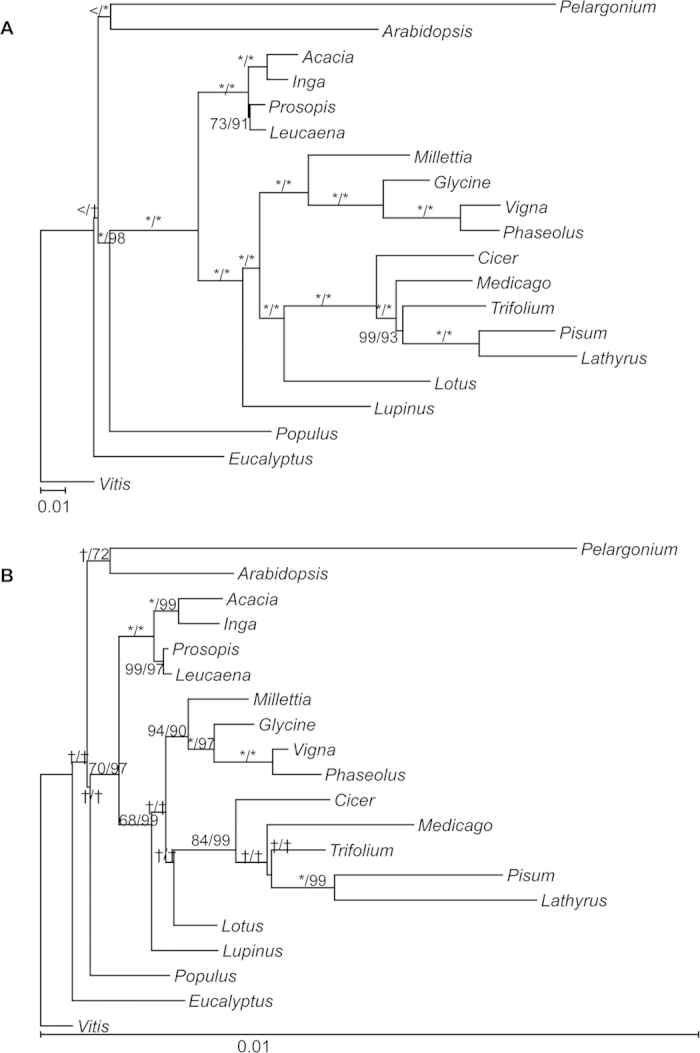
Relative rates of change for the substitution- and indel-only matrices. Branch lengths were estimated using a combined matrix with 74 protein coding genes that were retained and alignable across at least 18 of 20 taxa. Branch lengths were calculated with RAxML^70^ using a tree based on the current understanding of relationships for these taxa. (**A**) Substitution-only matrix with branch lengths estimated using GTRGAMMA. (**B**) Indel-only matrix treating indels as binary characters using BINGAMMA. Maximum likelihood and parsimony bootstraps are given above each node (“MLB/PB”), with an “*”, “>”, and an “†” indicating 100% support, < 50% support, “not applicable”, respectively. Scales are substitutions per site.

With the prevalence of plastome size expansion due to tandem repeat expansion in mimosoids and the idea that protein coding rate shifts may be associated with changes in DNA replication, repair, and/or recombination mechanisms, we investigated whether increased length related mutation rates in non-coding regions carried over into protein coding genes. Using established phylogenetic relationships for rosids^25^ and Leguminosae^3^, we calculated branch lengths and mean substitution rates (Table 2) for substitution-only (Fig. 6A) and indel-only matrices (Fig. 6B), using 74 retained and alignable protein coding genes. A comparison of the rates calculated from the original data file to 500 bootstrap replicate runs (e.g., mimosoid rate compared to mimosoid bootstrap rate) did not find a significant difference between the two for any clade (Table 2).

The substitution-only results (Table 2, Fig. 6A) are consistent with recent estimates of substitution rates and branch lengths for papilionoid plastomes^6^,^27^, as well as broad patterns for mimosoid taxa for the plastid genes *rbcL* and *matK*^55^. Our tree is also characterized by short branches and low substitution rates in mimosoids (mean 4.83 × 10^−4^ subst./site/Ma) and notably longer (3.1X) branches and higher substitution rates in papilionoids (mean 1.52  × 10^−3^ subst./site/Ma) (Fig. 6A). The IRLC lineages were the most rapidly evolving (ca. 3.56X the mimosoid rate). This pattern is striking and will likely hold up with increased sampling of legume plastomes (Koenen *et al.* unpubl. data). The underlying causes of this rate variation remain unknown, but it is perhaps notable that mimosoids are almost all woody tropical perennials whereas the phaseoloid and IRLC papilionoids are predominantly annual or short-lived herbaceous plants. The impacts of these apparently large punctuated shifts in plastome substitution rates across legumes for estimating divergence times are clearly apparent^55^.

Relative to the substitution tree, the indel tree (Table 2, Fig. 6B) shows noticeably lower rates of mutation on a per site basis (3.6%). Within that scaled context, the primary mimosoid versus general legume and papilionoid patterns mirror those from the substitution-only matrix. The indel tree has comparatively longer (2.95X) papilionoid branches (mean 5.6 × 10^−5^ subst./site/Ma) than mimosoid branches (mean 1.9 × 10^−5^ subst./site/Ma). Thus, indel related events that are influencing the size of mimosoid plastomes do not appear to have an obvious impact on patterns of substitution or indels in the corresponding protein coding regions.

However, the rates of indel variation across the sample of papilionoid plastomes revealed considerable among-lineage variation, with the mean IRLC rate being 2.2X that of the phaseoloid rate (Table 2). The same comparison in the substitution tree recovered only a 1.46X increase. Thus variation in indel rate among papilionoids appears to be greater than substitution-only variation, identifying a need to investigate the different causes of substitution and indel related mutation among these lineages.

## Conclusions

The addition and comparative analysis of new plastomes representing mimosoid legumes has provided valuable new insights into legume plastome variation. Unlike most papilionoids, mimosoid plastomes share the overall structure and gene content of the ancestral angiosperm plastome, but, like other sampled legumes, they have lost *rpl22*. Relative to the non-legume rosids and legume plastomes, tandem repeat expansion in mimosoids has led to substantial increases in overall plastome size. In *Acacia* and *Inga*, a large IR expansion into the SSC region, with just four genes remaining in the SSC, has further contributed to these being the largest known legume plastomes. *Acacia* also harbors a small LSC/IR shift. Rates of substitution and indel-associated mutation in mimosoid protein coding genes are low relative to papilionoid plastomes, where considerable rate variation was observed with regard to indels. However, rate variation observed in a subset of genes (e.g., *clpP*) adds to a growing body of knowledge on correlated rate changes among divergent angiosperm lineages, further hinting at shared common mechanisms. Our findings highlight the need for wider sampling of legume plastomes, especially across caesalpinioids and early-branching papilionoids, to ascertain the evolutionary history and extent of these plastid rearrangements across legumes as a whole, while at the same time contributing to larger phylogenetic data sets that are needed to generate a more robust legume phylogeny^28^ (and Koenen *et al.* in prep.). These advances in our understanding of legume plastome evolution provide important new resources for legume crop breeding studies and plastid genetic engineering of these economically important lineages.

## Materials and Methods

### DNA Extraction, Sequencing, and Assembly

#### Inga leiocalycina

DNA was extracted using the Sigma Chloroplast DNA Isolation kit (Cat. CPISO; Sigma, St. Louis, MO). An Illumina 400 bp insert TruSeq V2 library (Illumina, San Diego, CA) was sequenced (2 × 100 bp on ⅛ of lane) on a HiSeq 2000 with Macrogen (www.macrogen.com). High quality reads were filtered and trimmed using Trimmomatic V0.32^56^: ILLUMINACLIP:<adapters.fa>:2:30:10:8:TRUE MAXINFO:40:0.1 LEADING:20 TRAILING:20.

The assembly process employed Velvet (v1.2.10)^57^ to develop the primary assembly and contigs from ABySS (v1.3.4)^58^ and SOAPdenovo (v1.05)^59^ to help fill in gaps. A variety of kmer values were employed (39–64). Contigs were mapped to the *Millettia pinnata* reference. Reads were mapped back to this sequence with Bowtie^60^ to assess coverage and to correct errors. When allowing reads to map twice (−k 2), the inverted repeat should have twice the estimated coverage as single-copy regions. Coverage for different regions was estimated with genomeCoverageBed in BEDtools^61^. 2x higher coverage was apparent for the inverted repeat regions as well as part of the SSC. We hypothesized that the SSC genes with 2x coverage had become duplicated in the IR. By inspecting reads that map at the SSC/IR boundaries, we were able to establish the most probable boundaries. The annotated plastome sequence has been deposited in GenBank (KT428296) and the Illumina reads are in SRA307980.

#### Leucaena trichandra

DNA isolation followed a modified version of Georgi *et al.*^62^. An Illumina 300 bp insert TruSeq V2 library was prepared and sequenced by the Center for Genome Research at Oregon State University (http://cgrb.oregonstate.edu/) and sequenced on a HiSeq 2000 (2 × 100 bp sequencing on ⅓ of a lane). High quality reads were filtered and trimmed using Trimmomatic V0.34^56^ using: ILLUMINACLIP:2:30:10 and LEADING:25 TRAILING:25 SLIDINGWINDOW:5:25 MINLEN:65.

A linear plastome assembly including just one IR was generated using the Geneious v6.1.6 (Biomatters, Auckland NZ) as the *de novo* assembler and 3 M read-pairs of data (ca. 100X coverage). Subsequently, all 30 M read-pairs were mapped to the genome to correct errors and confirm the full genomic sequence. Independently assembled *de novo* IR boundary regions were reciprocally mapped to draft IR boundaries to confirm draft boundaries. These were confirmed using the BLAST-on-BLAST method discussed below (see “dispersed repeats”). The structural organization of the plastome assembly has since been confirmed by the addition of a 4 kb insert Illumina Nextera mate pair library developed and sequenced (2 × 100 bp reads) by the University of Maryland’s Genomic Resource Center. The annotated plastome sequence has been deposited in GenBank (KT428297) and the Illumina reads are in SRA305491.

### Annotations

Primary annotations involved Dogma^63^, confirmation with the *Glycine max* reference (NC_007942), open reading frame confirmation for protein coding genes using Geneious, and tRNA boundary confirmation/correction using the tRNAscan web interface with default settings (http://lowelab.ucsc.edu/tRNAscan-SE/)^64^.

### Interspecific Comparisons

All comparative analyses included the available published (as of March 2015) legume plastomes (one per species when multiple were available) plus closely related rosid plastomes that incorporate much of the known variation within those lineages. Species and NCBI Reference identifiers are in listed Table 1.

### Repeat Analyses

The total number of repeats, total base pairs in repeats, and percentage of the genome occupied by repeats were compared across species for mononucleotide (>8 bp), tandem, and dispersed repeats (>16 bp). Mononucleotide repeats were characterized using an in-house R script. In short, the plastome FASTA files were individually read into R and split into 8-nt sliding windows. Each window was evaluated for a mononucleotide string. If such a window was found, additional ones were built onto it for as long as the strings continued. Once a window containing a new nucleotide was reached, the script recorded the location, length, and mononucleotide string [repeat]. Tandem repeat composition and distribution were identified using the Tandem Repeat Finder web interface^65^ using default settings. We employed a BLAST-on-BLAST^66^ approach to identify dispersed repeats longer than 16 bp. Each plastome was searched against itself using “-word_size 16” and a 95% similarity cutoff.

### Protein Coding Rate Variation

Using a phylogeny based on previously established relationships among relevant rosids^25^ and Leguminosae taxa^3^, we calculated the branch lengths for two data sets, one based on substitution characters only (“substitution matrix”) and one based on indel characters only (“indel matrix”). Each data set included all 20 plastomes (Table 1) and all readily alignable protein coding genes found in at least 18 of those plastomes (74 genes). Protein coding sequence alignments were generated using MACSE^67^, with options “-prog alignSequences -gc_def 11 –gap_op 1”. Characters for simple indel coding were generated using gapcoder.py^68^. Matrices were concatenated for the plastome-wide substitution-only and indel-only analyses using phyutility.jar^69^ and RAxML^70^ was used to generate branch lengths on the reference tree (using “raxmlHPC -f e -m GTRGAMMA -o Vi_vi” for substitutions and “raxmlHPC -f e -m BINGAMMA -o Vi_vi” for indels). Using data derived from Lavin *et al.*^71^, the age of the legume clade and relevant mimosoid subclade were set to 59 and 33.2 Ma, respectively. Estimates for the mean rate of change for the legume, mimosoid, papilionoid, milletioid, and IRLC clades were calculated using r8s^72^ on the best fitting ML tree (see above). The mean value for each group was tested against the mean value of 500 bootstrap replicated matrices (generated using an in house R script) using a t-test. The *dN/dS* trees and ratios were calculated using the reading frame constrained MASCE^67^ alignments and the F3 × 4 model employed in PAML’s (v4.7) codeml^49^.

## Additional Information

**How to cite this article**: Dugas, D. V. *et al.* Mimosoid legume plastome evolution: IR expansion, tandem repeat expansions, and accelerated rate of evolution in *clpP.* *Sci. Rep.* **5**, 16958; doi: 10.1038/srep16958 (2015).

## Supplementary Material

Supplementary Information

## Figures and Tables

**Table 1:** Plastome characteristics.

	Species	NCBI	Total	LSC	SSC	IRs	GC %
Other Rosids	*Arabidopsis thaliana*	NC_000932	154478	84170	17780	26264	36.29
	*Eucalyptus grandis*	NC_014570	160137	88872	18475	26395	36.89
	*Pelargonium* x *hortorum*	NC_008454	217942	59710	6750	75741	39.61
	*Populus trichocarpa*	NC_009143	157033	85129	16600	27652	36.68
	*Vitis vinifera*	NC_007957	160928	89140	19082	26353	37.40
Mimosoideae	*Acacia ligulata*	LN555649.2	174233	92798	4985	38225	36.21
	*Inga leiocalycina*	KT428296	175489	90987	4948	39777	35.50
	*Leucaena trichandra*	KT428297	164692	93690	18890	26056	35.61
	*Prosopis glandulosa*	KJ_68101	163040	92322	18880	25919	35.86
Papilionoideae	*Cicer arietinum*	NC_011163	125319	NA	NA	NA	33.91
	*Glycine max*	NC_007942	152218	83175	17895	25574	35.37
	*Lathyrus sativus*	NC_014063	121020	NA	NA	NA	35.11
	*Lotus japonicus*	NC_002694	150519	81936	18271	25156	36.03
	*Lupinus luteus*	NC_023090	151894	82327	17847	25860	36.61
	*Medicago truncatula*	NC_003119	124033	NA	NA	NA	33.97
	*Millettia pinnata*	NC_016708	152968	83401	18511	25528	34.83
	*Phaseolus vulgaris*	NC_009259	150285	79823	17610	26426	35.44
	*Pisum sativum*	NC_014057	122169	NA	NA	NA	34.83
	*Vigna radiata*	NC_013843	151271	80898	17411	26481	35.23
	*Trifolium subterraneum*	NC_011828	144763	NA	NA	NA	34.4

NCBI accession number, total length of the plastome (bp), large single copy bp (LSC), inverted repeats bp (IR), and small single copy bp (SSC), as well as the percent GC content (%) for the complete plastome.

“NA” – refers to the IRLC legumes that lack the IR.

**Table 2:** Mean evolutionary rates.

	Substitution Matrix				Indel Matrix			
	Mean subst./site/MA	St. Dev.	Bootstrap Mean subst./site/MA	p-value	Mean subst./site/MA	St. Dev.	Bootstrap Mean subst./site/MA	p-value
Legume Clade	1.24E–03	5.06E–04	1.24E–03	9.97E–01	4.54E–05	3.51E–05	4.51E–05	9.76E–01
Mimosoid Clade	4.83E–04	1.21E–04	4.83E–04	9.97E–01	1.85E–05	1.17E–05	1.84E–05	9.92E–01
Papilionoid Clade	1.52E–03	2.45E–04	1.52E–03	9.92E–01	5.64E–05	3.48E–05	5.59E–05	9.67E–01
Phaseoloid Clade	1.45E–03	1.01E–04	1.45E–03	9.78E–01	3.76E–05	1.32E–05	3.68E–05	9.12E–01
IRLC	1.73E–03	1.48E–04	1.73E–03	9.98E–01	8.52E–05	3.44E–05	8.47E–05	9.78E–01

The mean evolutionary rate (subst./site/MA) of each major clade based on 74 protein coding genes calculated using r8s^72^. The first two columns for each matrix are calculations based on the original sequence matrix. The third column is the mean bootstrap value for 500 replicate runs derived from the original matrix. The fourth column is the p-vale for the t-test comparison between the rates for each group derived from the original matrix compared to the mean rate values for each group derived from the bootstrap matrices.
