# Supplementary material for: Mimosoid legume plastome evolution: IR expansion, tandem repeat expansions, and accelerated rate of evolution in clpP
Source: Sci Rep. 2015 Nov 23;5:16958. doi: 10.1038/srep16958 (PMC4655330; doi:10.1038/srep16958)
Supplement: Supplementary Information [file srep16958-s1.pdf]

Supplementary Materials.

Title: Mimosoid legume plastome evolution: IR expansion, tandem repeat expansions, and accelerated rate of evolution in *clpP*.

Authors: Diana V. Dugas, David Hernandez, Erik Koenen, Erika Schwarz, Shannon Straub, Colin E. Hughes, Robert K. Jansen, Madhugiri Nageswara-Rao, Martijn Staats, Joshua Trujillo, Nahid H. Hajrah, Njud S. Alharbi, Abdulrahman L. Al-Malki, Jamal S. M. Sabir, and C. Donovan Bailey

Supplementary Figure 1. Trees with *dN/dS* ratios calculated in PAML v4.7[49]. A) *atpF*, B) *clpP* C) *cemA*, D) *psbH*, E) *psbT*, F) *rps2*, G) *rps3*, and H) *rps4*. Values given for each node or terminal represent the dN/dS ratio. An “*” indicates cases with a *dS* of 0 and *dN* > 0.

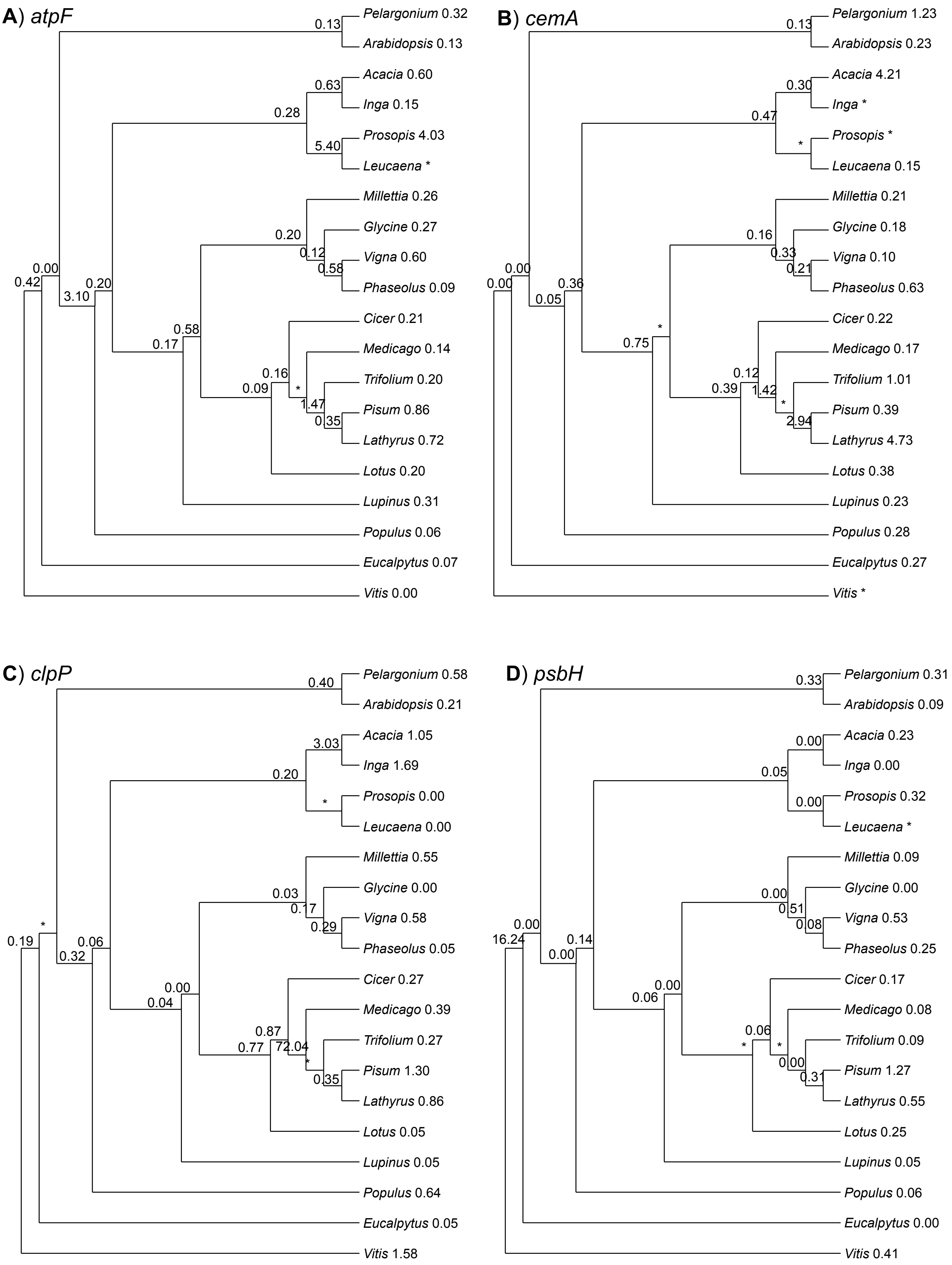

A) atpF
B) cemA
C) clpP
D) psbH
Pelargonium 0.32
Arabidopsis 0.13
Acacia 0.60
Inga 0.15
Prosopis 4.03
Leucaena *
Millettia 0.26
Glycine 0.27
Vigna 0.60
Phaseolus 0.09
Cicer 0.21
Medicago 0.14
Trifolium 0.20
Pisum 0.86
Lathyrus 0.72
Lotus 0.20
Lupinus 0.31
Populus 0.06
Eucalpytus 0.07
Vitis 0.00
Pelargonium 1.23
Arabidopsis 0.23
Acacia 4.21
Inga *
Prosopis *
Leucaena 0.15
Millettia 0.21
Glycine 0.18
Vigna 0.10
Phaseolus 0.63
Cicer 0.22
Medicago 0.17
Trifolium 1.01
Pisum 0.39
Lathyrus 4.73
Lotus 0.38
Lupinus 0.23
Populus 0.28
Eucalpytus 0.27
Vitis *
Pelargonium 0.58
Arabidopsis 0.21
Acacia 1.05
Inga 1.69
Prosopis 0.00
Leucaena 0.00
Millettia 0.55
Glycine 0.00
Vigna 0.58
Phaseolus 0.05
Cicer 0.27
Medicago 0.39
Trifolium 0.27
Pisum 1.30
Lathyrus 0.86
Lotus 0.05
Lupinus 0.05
Populus 0.64
Eucalpytus 0.05
Vitis 1.58
Pelargonium 0.31
Arabidopsis 0.09
Acacia 0.23
Inga 0.00
Prosopis 0.32
Leucaena *
Millettia 0.09
Glycine 0.00
Vigna 0.53
Phaseolus 0.25
Cicer 0.17
Medicago 0.08
Trifolium 0.09
Pisum 1.27
Lathyrus 0.55
Lotus 0.25
Lupinus 0.05
Populus 0.06
Eucalpytus 0.00
Vitis 0.41

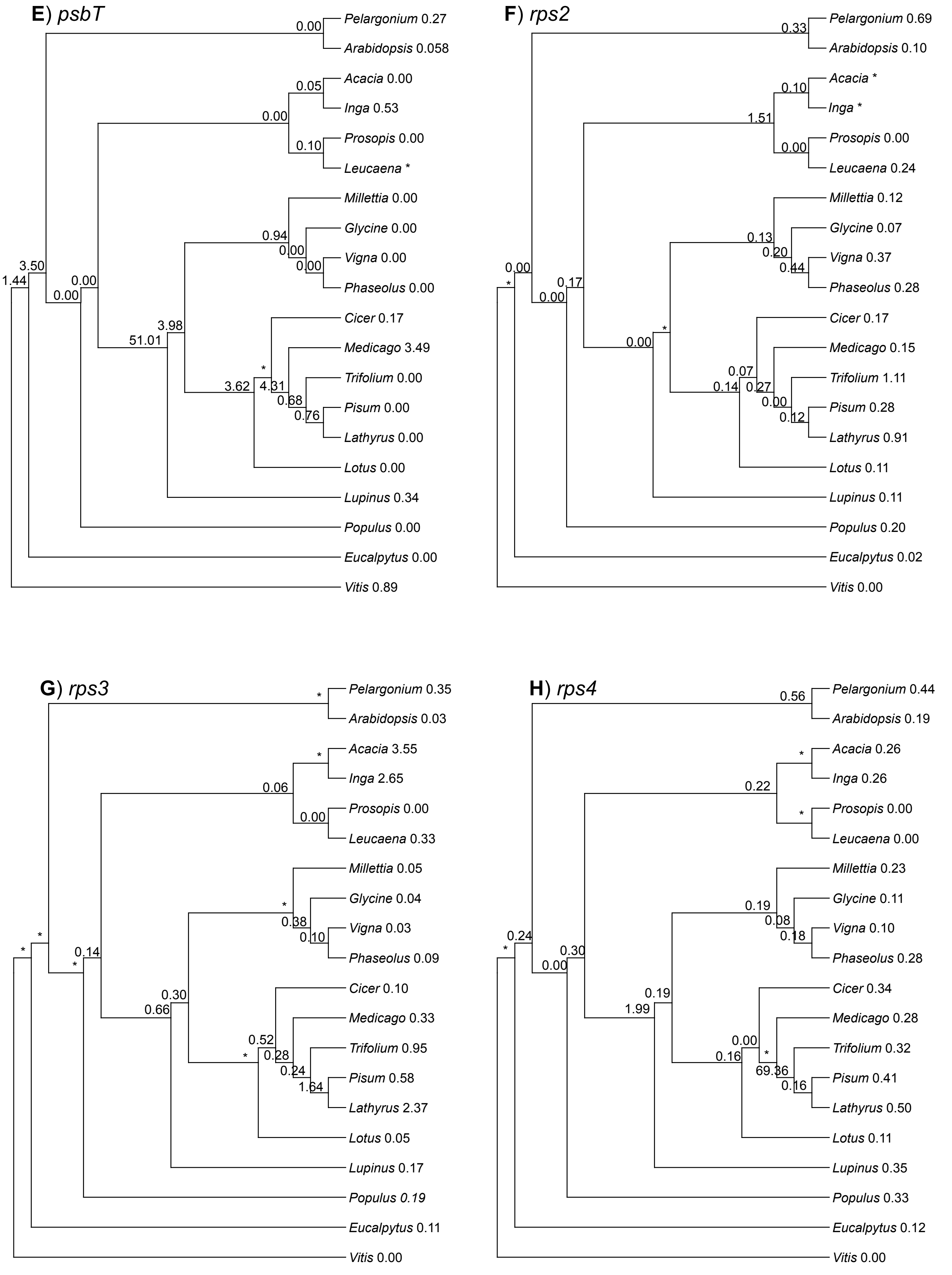
E) psbT
Pelargonium 0.27
Arabidopsis 0.058
Acacia 0.00
Inga 0.53
Prosopis 0.00
Leucaena *
Millettia 0.00
Glycine 0.00
Vigna 0.00
Phaseolus 0.00
Cicer 0.17
Medicago 3.49
Trifolium 0.00
Pisum 0.00
Lathyrus 0.00
Lotus 0.00
Lupinus 0.34
Populus 0.00
Eucalpytus 0.00
Vitis 0.89
0.00
0.05
0.00
0.10
0.94
0.00
0.00
3.50
1.44
0.00
0.00
3.98
51.01
3.62
*
4.31
0.68
0.76
F) rps2
Pelargonium 0.69
Arabidopsis 0.10
Acacia *
Inga *
Prosopis 0.00
Leucaena 0.24
Millettia 0.12
Glycine 0.07
Vigna 0.37
Phaseolus 0.28
Cicer 0.17
Medicago 0.15
Trifolium 1.11
Pisum 0.28
Lathyrus 0.91
Lotus 0.11
Lupinus 0.11
Populus 0.20
Eucalpytus 0.02
Vitis 0.00
0.33
0.10
1.51
0.00
0.13
0.20
0.44
0.00
*
0.17
0.00
*
0.00
0.07
0.14
0.27
0.00
0.12
G) rps3
Pelargonium 0.35
Arabidopsis 0.03
Acacia 3.55
Inga 2.65
Prosopis 0.00
Leucaena 0.33
Millettia 0.05
Glycine 0.04
Vigna 0.03
Phaseolus 0.09
Cicer 0.10
Medicago 0.33
Trifolium 0.95
Pisum 0.58
Lathyrus 2.37
Lotus 0.05
Lupinus 0.17
Populus 0.19
Eucalpytus 0.11
Vitis 0.00
*
*
0.06
0.00
*
0.38
0.10
*
*
0.14
*
0.30
0.66
0.52
0.28
0.24
1.64
*
H) rps4
Pelargonium 0.44
Arabidopsis 0.19
Acacia 0.26
Inga 0.26
Prosopis 0.00
Leucaena 0.00
Millettia 0.23
Glycine 0.11
Vigna 0.10
Phaseolus 0.28
Cicer 0.34
Medicago 0.28
Trifolium 0.32
Pisum 0.41
Lathyrus 0.50
Lotus 0.11
Lupinus 0.35
Populus 0.33
Eucalpytus 0.12
Vitis 0.00
0.56
*
0.22
*
0.19
0.08
0.18
0.24
*
0.30
0.00
0.19
1.99
0.00
0.16
*
69.36
0.16
